# Early Recognition of Subcutaneous Fat Necrosis of the Newborn With Atypical Purpuric Presentation

**DOI:** 10.7759/cureus.89938

**Published:** 2025-08-12

**Authors:** Eri Ohta, Kayo Yamamoto, Natsuko Saito-Sasaki, Etsuko Okada, Yu Sawada

**Affiliations:** 1 Dermatology, University of Occupational and Environmental Health, Kitakyushu, JPN

**Keywords:** case report, dermoscopy, histology, skin, subcutaneous fat necrosis of the newborn

## Abstract

Subcutaneous fat necrosis of the newborn (SCFN) is a rare panniculitis that typically presents within the first few weeks of life. We report a unique case of SCFN diagnosed as early as day three of life in a large-for-gestational-age neonate born to a diabetic mother. Notably, the lesion exhibited a central purpuric area with an irregular, serrated erythematous border, which was clearly visualized using dermoscopy. Histological confirmation was limited due to sampling constraints, but the clinical features and evolution were consistent with SCFN. This case highlights the utility of early dermatologic assessment and dermoscopy in identifying SCFN with atypical hemorrhagic features, expanding the clinical spectrum of this condition.

## Introduction

Subcutaneous fat necrosis of the newborn (SCFN) is an uncommon inflammatory disorder affecting term or post-term neonates [1]. It usually presents around the first week of life as firm, erythematous plaques localized on pressure-prone areas, such as the back, shoulders, and buttocks [2,3]. While the diagnosis is primarily clinical, histopathological confirmation is often used, particularly in atypical cases [2]. Dermoscopy, though widely used in dermatology, has rarely been applied to SCFN.

We present a rare case of SCFN that manifested with a central purpuric plaque and an irregular erythematous margin as early as day three postpartum. This report is the first to describe detailed dermoscopic findings in SCFN, including hemorrhagic visualization and irregular margins.

## Case presentation

A female neonate was delivered at 37 weeks and two days of gestation via elective cesarean section. The mother had a history of type 2 diabetes mellitus and previous placental abruption. The birth weight was 3,882 g (above the 99th percentile), classifying the infant as large-for-gestational-age (LGA). Apgar scores were 8 and 9 at one minute and five minutes, respectively. At birth, peripheral cyanosis was noted, and oxygen therapy was initiated. Within the first hour of life, respiratory distress progressed, requiring NICU admission and intubation. Initial laboratory evaluation on the day of birth revealed the following values: representative parameters included white blood cell count of 13.2 × 10^3^/μL and segmented neutrophils of 64%. C-reactive protein was <0.01 mg/dL.

**Figure 1:**
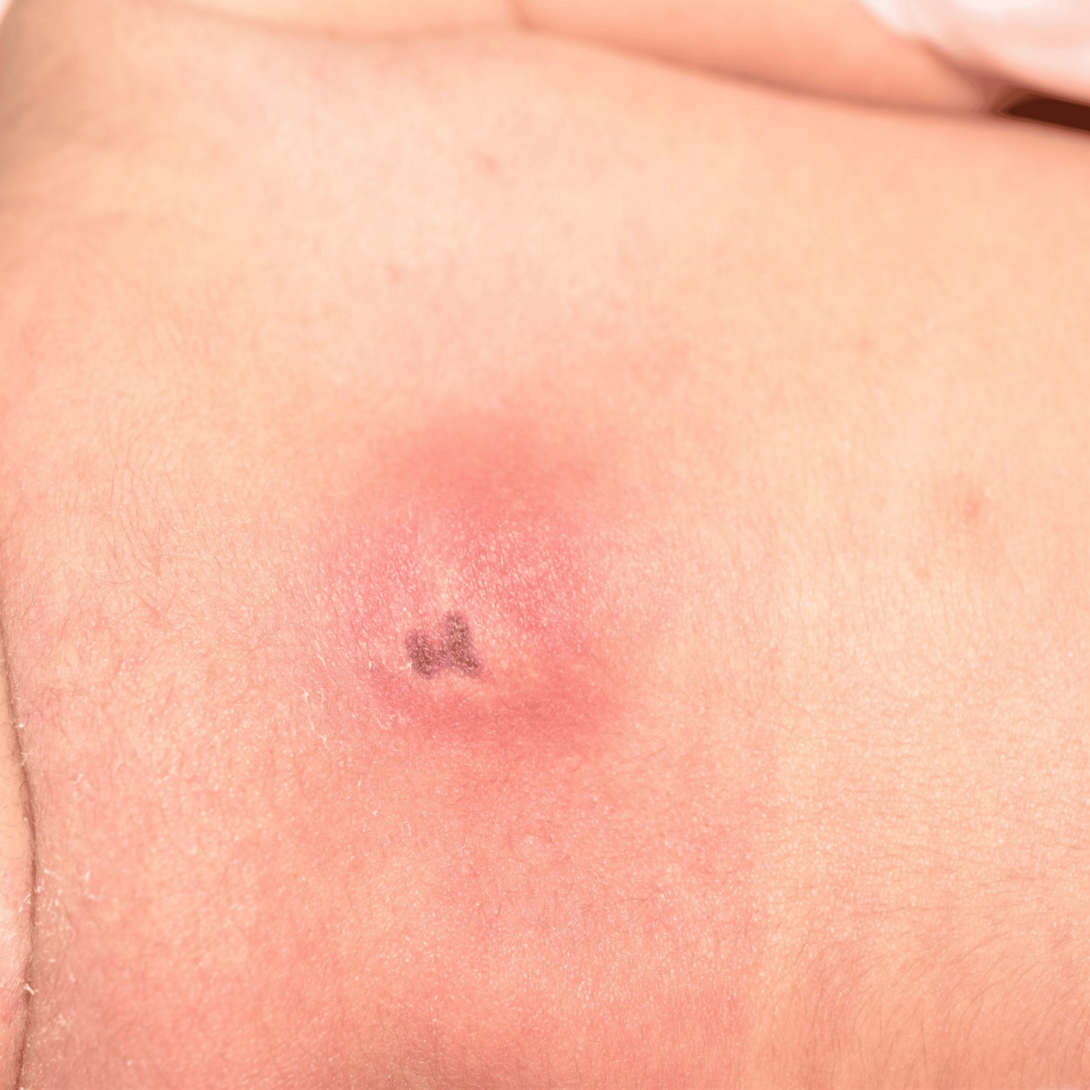
Early clinical presentation of subcutaneous fat necrosis of the newborn A firm, violaceous plaque with central darkening on the upper back is visible on day three of life. The lesion measures approximately 2.5 cm in diameter and appears indurated but non-tender, consistent with early subcutaneous fat necrosis of the newborn (SCFN).

**Figure 2:**
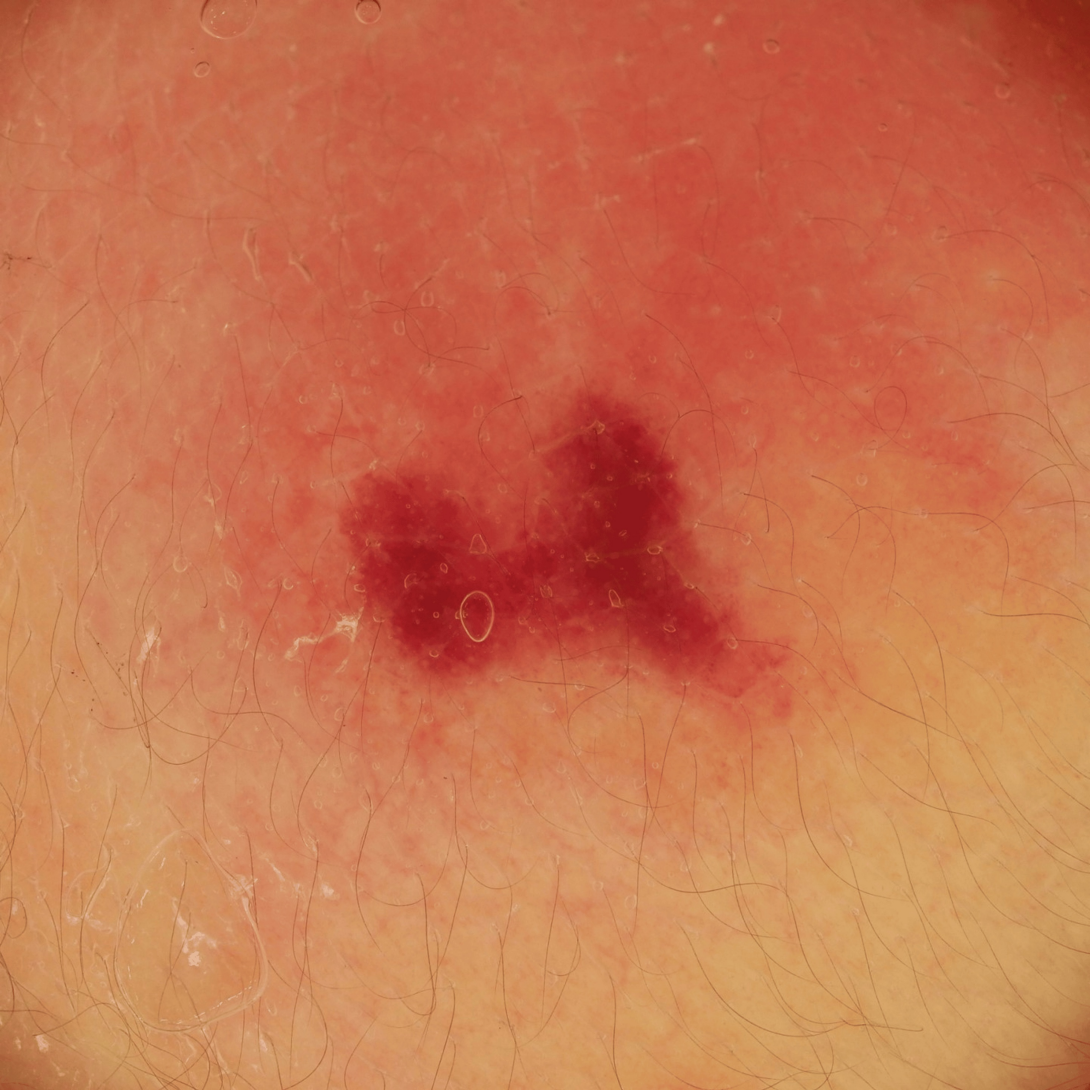
Dermoscopic features of the purpuric lesion Dermoscopy reveals a central purpuric zone with an irregular, serrated erythematous border. Fine vascular disruption and perifocal pallor are also noted. These features are not typically described in subcutaneous fat necrosis of the newborn (SCFN) and may represent early hemorrhagic and inflammatory changes.

On day three, during extubation and routine examination, a firm, violaceous plaque was observed on the upper back (Figure 1). The lesion was non-tender, indurated, and measured approximately 2.5 cm in diameter. Dermoscopic evaluation revealed a central dark reddish-purpuric zone surrounded by an irregular, serrated erythematous margin. The border was non-uniform, lacking symmetry, with fine vascular disruption and peripheral pallor (Figure 2).

**Figure 3:**
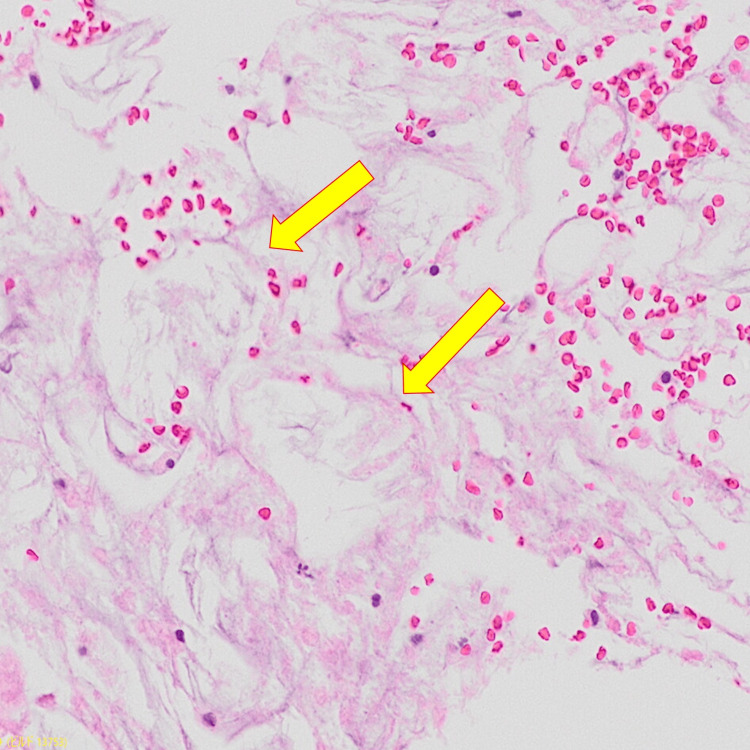
Histopathologic findings of fat necrosis Histological section of subcutaneous tissue shows red blood cell extravasation and needle-shaped clefts within adipocytes (yellow arrows), suggesting crystal deposition.

A punch biopsy was attempted; however, due to the hemorrhagic nature of the lesion, only deep subcutaneous adipose tissue was obtained. Histopathologic examination revealed hemorrhage between fat lobules. Importantly, several adipocytes exhibited radiating and needle-shaped clefts within their cytoplasm, features consistent with crystal deposition (Figure 3).

**Figure 4:**
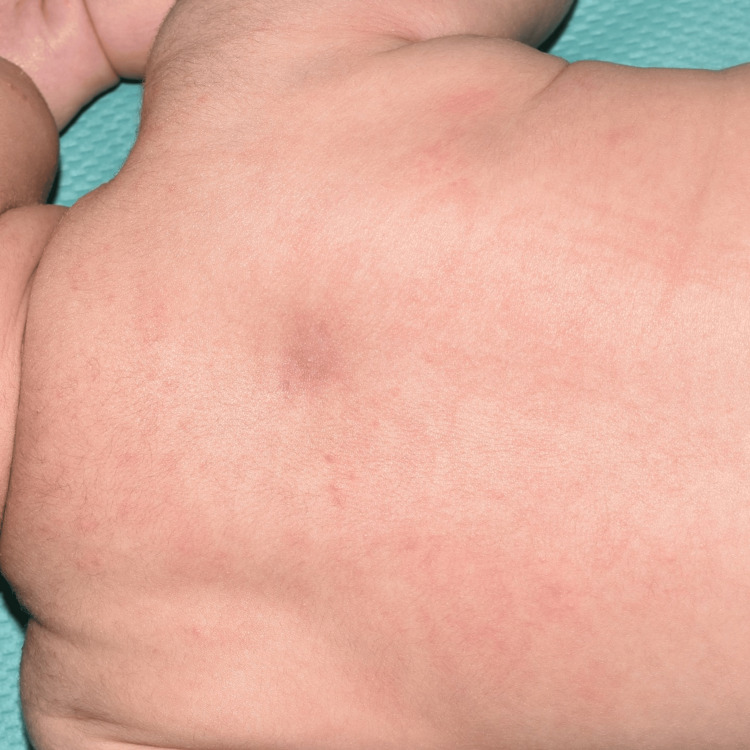
Resolution phase of the lesion At day 42, the lesion shows near-complete resolution with faint post-inflammatory hyperpigmentation and a small biopsy scar. This clinical course is consistent with the self-limiting nature of subcutaneous fat necrosis of the newborn (SCFN).

By day 42 of life, only faint post-inflammatory hyperpigmentation and a small biopsy scar remained at the lesion site, indicating nearly complete resolution (Figure 4).

## Discussion

SCFN is typically associated with perinatal stress, such as hypoxia, hypothermia, or maternal diabetes [4-6]. Our case was notable for three key features: extremely early onset (day three of life), presence of a central purpuric plaque with dermoscopically visible serrated erythematous borders, and histologic inconclusiveness due to hemorrhage.

While most SCFN cases present around the one week of life [7], this patient developed visible cutaneous findings within 72 hours of birth. This early onset, especially in the context of maternal diabetes and LGA status, underscores the importance of close dermatologic surveillance in high-risk neonates.

The dermoscopic identification of a central purpuric zone with irregular borders appears to be novel in SCFN. While erythematous plaques are common in SCFN, the coexistence of well-demarcated purpura is atypical and clinically significant. To our knowledge, no previous studies have systematically described purpuric morphology in SCFN, nor provided dermoscopic analysis of such hemorrhagic features [7]. In this case, dermoscopy enabled real-time visualization of both a central hemorrhagic zone and a peripheral erythematous rim with serrated margins. This contrast may reflect perifocal inflammation and early fat necrosis, along with subcutaneous hemorrhage. Although histopathology demonstrated red blood cell extravasation within adipose tissue, definitive evidence of capillary wall damage or vascular disruption was not observed in the specimen. The sharply defined purpura and irregular erythema appear as separate yet overlapping components, offering valuable diagnostic insight. These changes are likely more apparent in neonates, whose skin is thinner and vasculature more fragile than in older children or adults [8].

## Conclusions

This case underscores the importance of considering SCFN in neonates presenting with atypical purpuric or violaceous skin lesions, which may mimic infectious or vascular etiologies. The use of dermoscopy provided valuable, noninvasive insight into the vascular and inflammatory features of SCFN, including central purpura and irregular peripheral borders, which may reflect underlying fat necrosis and perifocal inflammation. As neonatal skin is thin and histologic evaluation can be limited due to clinical limitations, dermoscopy may serve as an adjunctive tool to enhance diagnostic confidence and guide timely management. Further systematic accumulation and characterization of dermoscopic findings in neonatal panniculitis are essential to establish standardized diagnostic criteria and improve early recognition in clinical practice.
